# Smartphone Apps for Smoking Cessation: Systematic Framework for App Review and Analysis

**DOI:** 10.2196/45183

**Published:** 2023-07-13

**Authors:** Krysten W Bold, Kathleen A Garrison, Angela DeLucia, Mark Horvath, Milton Nguyen, Erica Camacho, John Torous

**Affiliations:** 1 Department of Psychiatry Yale School of Medicine New Haven, CT United States; 2 Department of Psychiatry Beth Israel Deaconess Medical Center Boston, MA United States

**Keywords:** addict, app review, application, apps, cessation, cigarette, digital health, mhealth, mobile app, mobile health, quit, review of app, smartphone app, smoker, smoking cessation, smoking, tobacco

## Abstract

**Background:**

Cigarette smoking is a leading cause of preventable death, and identifying novel treatment approaches to promote smoking cessation is critical for improving public health. With the rise of digital health and mobile apps, these tools offer potential opportunities to address smoking cessation, yet the functionality of these apps and whether they offer scientifically based support for smoking cessation are unknown.

**Objective:**

The goal of this research was to use the American Psychiatric Association app evaluation model to evaluate the top-returned apps from Android and Apple app store platforms related to smoking cessation and investigate the common app features available for end users.

**Methods:**

We conducted a search of both Android and iOS app stores in July 2021 for apps related to the keywords “smoking,” “tobacco,” “smoke,” and “cigarette” to evaluate apps for smoking cessation. Apps were screened for relevance, and trained raters identified and analyzed features, including accessibility (ie, cost), privacy, clinical foundation, and features of the apps, using a systematic framework of 105 objective questions from the American Psychiatric Association app evaluation model. All app rating data were deposited in mindapps, a publicly accessible database that is continuously updated every 6 months given the dynamic nature of apps available in the marketplace. We characterized apps available in July 2021 and November 2022.

**Results:**

We initially identified 389 apps, excluded 161 due to irrelevance and nonfunctioning, and rated 228, including 152 available for Android platforms and 120 available for iOS platforms. Some of the top-returned apps (71/228, 31%) in 2021 were no longer functioning in 2022. Our analysis of rated apps revealed limitations in accessibility and features. While most apps (179/228, 78%) were free to download, over half had costs associated with in-app purchases or full use. Less than 65% (149/228) had a privacy policy addressing the data collected in the app. In terms of intervention features, more than 56% (128/228) of apps allowed the user to set and check in on goals, and more than 46% (106/228) of them provided psychoeducation, although few apps provided evidence-based support for smoking cessation, such as peer support or skill training, including mindfulness and deep breathing, and even fewer provided evidence-based interventions, such as acceptance and commitment therapy or cognitive behavioral therapy. Only 12 apps in 2021 and 11 in 2022 had published studies supporting the feasibility or efficacy for smoking cessation.

**Conclusions:**

Numerous smoking cessation apps were identified, but analysis revealed limitations, including high rates of irrelevant and nonfunctioning apps, high rates of turnover, and few apps providing evidence-based support for smoking cessation. Thus, it may be challenging for consumers to identify relevant, evidence-based apps to support smoking cessation in the app store, and a comprehensive evaluation system of mental health apps is critically important.

## Introduction

Cigarette smoking remains the leading cause of preventable death worldwide [1-3]. In the United States alone, approximately 34.1 million adults currently smoke cigarettes [2]. Over 55% of adults who smoke try to quit each year; however, most adults try to quit entirely on their own without the help of medication or formal counseling, and very few succeed in quitting long-term [3]. Reasons for low use of standard smoking cessation interventions include barriers such as limited access, stigma, cost, provider availability, and training [4-8].

Delivering smoking cessation interventions through mobile technologies and smartphone apps is one way to overcome these barriers and provide in-the-moment help to adults who want to quit smoking [8-11]. According to recent reports, 85% of US adults own a smartphone [12], and an estimated 80% of US adults who smoke and are motivated to quit own a smartphone [13]. Smoking cessation treatment delivered through smartphone apps has the potential to disseminate accessible, scalable, and cost-effective tools for smoking cessation [14,15]. For example, these apps can deliver important evidence-based behavioral components of smoking cessation interventions in a real-world environment, such as self-monitoring to identify smoking triggers and skill training to cope with craving.

There have been several promising studies of mobile smoking cessation interventions, including text message and app-based programs [11,16,17], underscoring the potential of mobile technology for promoting smoking cessation. In terms of smoking cessation apps, some studies have shown effectiveness for smoking cessation, such as the iCanQuit app [16,18]; however, overall, a Cochrane review found no evidence that smartphone apps increased the likelihood of smoking cessation in a meta-analysis across studies (risk ratio 1.00, 95% CI 0.66-1.52; *I*^2^=59%; 5 studies, 3079 participants) [17,19] and emphasized a need for more high-quality clinical trials.

The observed heterogeneity in efficacy across meta-analyses may be due to differences in app content and features. For example, a recent review found that while top smoking apps typically include relevant smoking cessation content related to developing a quit plan and enhancing motivation by describing the rewards of not smoking [20], another review found that many smoking cessation apps show limited adherence to clinical practice guidelines, such as connecting to a quit line or endorsing the use of quit smoking medications [21-23]. Notably, apps with lower adherence to clinical practice guidelines are often the most popular [21] and quit smoking apps available in the app store tend to lack scientific evidence [9,24], suggesting the end user of these apps may have a difficult time identifying an effective app to quit smoking. For example, in 1 review of the top 50 apps in app stores, only 2 had any scientific support [9]. There may be limited widespread benefit for promoting smoking cessation with smartphone apps if common apps found in the app store by the end user lack scientific support or fail to use evidence-based behavioral change techniques.

Using a systematic framework for evaluating apps may be one important tool for consumers to evaluate apps among the multitude of options that proliferate on the market. Recent efforts to standardize the evaluation of app usability (including engagement, functionality, and quality) include the Mobile App Rating Scale (MARS; [25]), user version of the Mobile App Rating Scale (uMARS; [26]), and the System Usability Scale [27,28]. While most studies report high feasibility and acceptability of apps when tested [8], few apps have been evaluated for quality and effectiveness [29] and most app rating systems are not available to the end user to help inform app selection. The aim of this paper is to use a standardized framework based on the American Psychiatric Association’s (APA) app evaluation model for informed decision-making regarding smoking cessation app evaluation that is available to anyone with a smartphone and internet connection [30,31]. The APA model of app evaluation has been widely studied and used, including evaluating apps for a number of conditions, including bipolar disorder [32], suicide prevention, and depression [33]. Thus, the same framework can be applied in a standardized way across a variety of apps related to mental health and behavioral health. Other benefits of this model include that the framework has been translated into a publicly accessible database format consisting of 105 objective questions to assess multiple domains, including functionality and accessibility, privacy and security, clinical foundation, and app engagement style (see Lagan et al [31]). Each question in the framework is derived from a principle in the APA app evaluation model and is coded as an objective, reproducible data point that can be used to evaluate and compare apps through a publicly available database (on mindapps [34]). A systematic framework that is publicly available to consumers may be especially important since the Food and Drug Administration recently stopped the software precertification pilot program that was intended to explore ways to evaluate digital health tools [35]. Thus, the goal of this research was to apply this APA app evaluation framework to the top-returned apps from Android and Apple app store platforms related to smoking cessation to investigate the common app features available for end users. The goal is not to evaluate or emphasize specific apps but rather to characterize common features in the top-returned apps readily available to a layperson using common search terms.

## Methods

### Overview

Apps were initially identified by searching app stores on the Android (Google Play Store) and Apple (iOS App Store) platforms in July 2021. Four search terms were selected based on common words related to smoking: “smoking,” “tobacco,” “cigarette,” and “smoke,” and we recorded at least the top 100 returned apps for each search term (n=222 “smoking,” n=146 “tobacco,” n=123 “cigarette,” and n=104 “smoke”). This app selection was meant to be reflective of a layperson using these search terms to find a relevant app. A total of 389 unique apps were returned across both the Android and iOS platforms. These 389 unique results were downloaded and reviewed following the procedures outlined below. Once apps are rated and entered into the mindapps database, each app is updated at least every 6 months to ensure that listed apps are functioning and to rerate the apps to reflect changes. Mindapps is a database derived from the APA app evaluation model that translates the principles of the APA model (eg, privacy, efficacy, engagement, and interoperability) into a searchable database comprised of 105 objective questions for each app. Every app in the database is reviewed and updated at least every 6 months. The mindapps database is open to the public and features videos and resources to help users select and evaluate apps.

### Training for App Review

The raters were trained to review app content to first determine relevant and irrelevant apps, excluding those that were not available in English and did not provide support or information relevant to quitting smoking (see “Rating Process and Data Analysis” for details on irrelevant apps). To review relevant apps, the raters were trained in using the app evaluation framework created from the APA’s App Evaluation Model. The 2 raters were trained to use this established rating framework by the training supervisor (author EC) who provided instruction about the rating system and provided practice apps for rating. The 2 raters independently rated 2 practice apps. Interrater reliability was assessed using the Cohen statistic. Good interrater reliability was demonstrated by the trained app raters, defined as a value above 0.7. Discrepancies were resolved in discussion between the raters and training supervisor (author EC) to ensure consistency in coding with practice apps before evaluating the smoking cessation apps.

### App Evaluation Framework

Apps were rated following an established framework, mindapps, based on the APA’s App Evaluation Model [36]. The rating framework involves 105 objective questions evaluating several features across 6 domains [37], described below. Each question in the framework is derived from a principle in the APA app evaluation model for mental health apps and is coded as an objective, reproducible data point that can be used to evaluate and compare apps [31]. These questions are standardized to be applicable across conditions and include features considered typical of mental health interventions (eg, tracking mood, symptoms, and cognitive behavioral therapy [CBT]). The framework has been used to evaluate apps for a number of conditions, including bipolar disorder [32], suicide prevention, and depression [33]. The rating system includes questions related to the following domains: (1) functionality and accessibility, such as available platforms (eg, iOS and Android), Spanish language option (yes/no), and cost (eg, free, subscription, and in-app purchases); (2) inputs and outputs, such as whether the app sends notifications or provides summaries of data to the user; (3) privacy and security, such as whether there is a privacy policy and whether personal health information or deidentified data are shared; (4) data sharing, such as whether data can be exported or shared by the user; (5) evidence and clinical foundation assessing whether there are any published feasibility, functionality, or efficacy studies about the app, based on searching peer-reviewed publication databases (eg, PubMed) for studies with the app name; and (6) features and engagement style that evaluate the intervention features available in the app, such as setting goals, learning psychoeducation, tracking mood and symptoms, learning CBT approaches, and engagement features, such as whether there are chat features, gamification, videos, or music.

### Rating Process and Data Analysis

**Figure 1:**
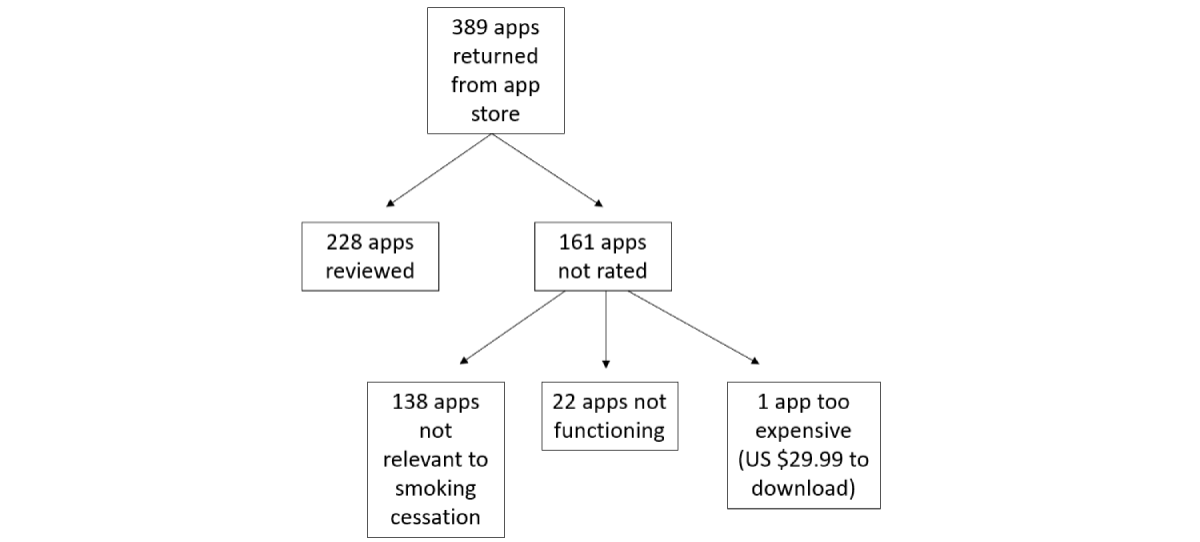
Flow diagram of top-returned apps for smoking cessation from Android and iOS stores collected in July 2021.

Of the 389 unique apps retrieved from the app stores in the initial app search in July 2021, a total of 161 were not rated due to irrelevance (n=138) or were nonfunctioning (n=22). A flow diagram is displayed in Figure 1. Raters confirmed that the included apps had smoking cessation content, and most of the excluded apps had content that was not related to quitting smoking, such as apps designed as digital cigarettes or lighters to simulate smoking, apps about web-based purchasing of products, or apps for finding places where smoking is allowed. Apps that were not free to download were purchased for analysis by raters if they were 10 dollars or less, or raters used free trials if provided by the app. Only 1 app out of 389 (0.25%) was not rated due to the high initial cost to download (US $29.99). The remaining 228 relevant apps were assigned to be reviewed by a trained rater who used each app for 20 minutes or longer until all rating criteria from the 105-question rating system were completed. Data entry was assessed and verified by the training supervisor (author EC) before being finalized in the mindapps database. A full list of rating questions is publicly available at mindapps and included in Multimedia Appendix 1. Additionally, the ratings for each individual app are included in the database of smoking cessation apps at mindapps website. This database is continuously updated to ensure that it only includes functioning apps and relevant updates are included (eg, newly published studies are noted to indicate support for the feasibility and efficacy of the app). Below, we outline the results from the apps that were originally identified and rated in the mindapps database related to smoking cessation in July 2021 and the current list available in November 2022. The resulting data were analyzed using descriptive statistics in SPSS (version 28; IBM Corp).

## Results

### Overview

Table 1 presents frequency information for common app characteristics. A total of 228 rated apps were entered in the database in July 2021, and 180 apps were available in November 2022. Apps were removed between 2021 and 2022 that were nonfunctioning (n=71) and 23 new apps were added and made available in November 2022. Multimedia Appendix 2 lists the names of apps rated and recorded in mindapps available in July 2021 and November 2022. The characteristics of apps were mostly consistent between those available in July 2021 and November 2022, except where differences are noted below.

**Table 1:** Characteristics of smoking cessation apps rated in July 2021 (N=228) and available in November 2022 (N=180).

Characteristics			Apps in 2021 (N=228), n (%)		Apps in 2022 (N=180), n (%)
**Platforms^a^**					
	iOS	120 (52.6)		103 (57.2)	
	Android	152 (66.7)		119 (66.1)	
	Both	44 (19.3)		42 (23.3)	
	Web	3 (1.3)		12 (6.7)	
**Functionality**					
	Spanish	56 (24.6)		39 (21.7)	
	Offline	176 (77.2)		147 (81.7)	
	Accessibility features	106 (46.5)		73 (40.6)	
	Crisis management feature	1 (0.4)		3 (1.7)	
**Cost**					
	Totally free	95 (41.7)		71 (39.4)	
	Free to download	179 (78.5)		149 (82.8)	
	In-app purchases	84 (36.8)		75 (41.7)	
	1-time payment	68 (29.8)		64 (35.6)	
	Subscription	36 (15.8)		36 (20)	
**Inputs**					
	Surveys	77 (33.8)		72 (40)	
	Diary	39 (17.1)		32 (17.8)	
	Camera	24 (10.5)		23 (12.8)	
	External devices	12 (5.3)		11 (6.1)	
	Social network	11 (4.8)		22 (12.2)	
	Geolocation	8 (3.5)		7 (3.9)	
	Microphone	3 (1.3)		18 (10)	
	Step count	2 (0.9)		0 (0)	
	Contact list	3 (1.3)		4 (2.2)	
**Outputs**					
	Provides references or information	152 (66.7)		100 (55.6)	
	Notifications	115 (50.4)		106 (58.9)	
	Data summary	154 (67.5)		140 (77.8)	
	Data graphs	100 (43.9)		82 (45.6)	
	Reminders	47 (20.6)		41 (22.8)	
	Social network posting	46 (20.2)		44 (24.4)	
**Privacy**					
	Has privacy policy	149 (65.4)		108 (60)	
	App clarifies data use and purpose	114 (50)		97 (53.9)	
	App reports data security measures	94 (41.2)		70 (38.9)	
	PHI^b^ is shared	82 (36)		67 (37.2)	
	Data stored on server	74 (32.5)		70 (38.9)	
	Data stored on device	22 (9.6)		22 (12.2)	
	Can opt out of data collection	24 (10.5)		17 (9.4)	
	De-identified data shared	22 (9.6)		23 (12.8)	
	Claims to meet HIPAA^c^	2 (0.9)		2 (1.1)	
**Data sharing**					
	Email or export data	50 (21.9)		45 (25)	
	User can see and access own data	39 (17.1)		42 (23.3)	
	Connect to medical record to share with providers	0 (0)		0 (0)	
**Evidence and clinical foundation**					
	Clinical claims	228 (100)		180 (100)	
	Clinically relevant	223 (97.8)		173 (96.1)	
	Clinical warning for use (eg, does not replace medical care)	19 (8.3)		23 (12.8)	
	Supporting studies	12 (5.2)		11 (6.1)	

^a^Platform represents the app store where the app was originally found as a top-returned app in the search. More apps may be available across both platforms.

^b^PHI: protected health information.

^c^HIPAA: Health Insurance Portability and Accountability Act.

### Functionality and Accessibility

#### Functionality

Assessment of functionality revealed that the majority of apps work offline (>77%, 176/228 in 2021 and 147/180 in 2022). Other accessibility features (eg, ability to adjust text size and text to voice) were only offered by less than half of the rated apps (<46%, 106/228 in 2021 and 73/180 in 2022). All apps were available in English and additional functionality to select Spanish-language versions was only available for less than 24% (56/228 in 2021 and 39/180 in 2022) of apps. Crisis management features (eg, a hotline number to call if in crisis) were very rarely available (<2%, 1/228 in 2021 and 3/180 in 2022).

#### Cost

Most apps (179/228, 78% in 2021 and 149/180, 82.7% in 2022) were free to download, but less than half of them were totally free. The average cost for apps requiring an up-front purchase to download in July 2021 was US $3.07 (SD 1.89; range 0.99-9.99). Over 58% (120/228 in 2021 and 111/180 in 2022) of the apps required some form of additional payment once downloaded, such as in-app purchases or subscriptions.

### Inputs and Outputs

The most common data inputs were surveys tracking information such as cigarette count, craving, or mood, followed by diary inputs that allow for free-writing or journaling. Less common inputs were connections to external devices, geolocation, and contact lists. A greater percentage of apps in 2022 received inputs from social networks (22/180, 12.2%) compared with 2021 (11/228, 4.8%). Common outputs from the app were notifications (eg, incoming messages and alerts), and many apps provided data summaries in the app or provided visual data graphs.

### Privacy and Security

Privacy policies were available for less than 65% (149/228 in 2021 and 108/180 in 2022) of apps. Review of the privacy policies revealed that only about 50% (114/228 in 2021 and 97/180 in 2022) of apps clarify the data use and purpose, <41% (94/228 in 2021 and 70/180 in 2022) report data security measures, and about 36% (82/228 in 2021 and 67/180 in 2022) of apps stated that they share personal health information that is entered into the app, such as name, birthday, and mental health information. Approximately 10% (24/228 in 2021 and 17/180 in 2022) or fewer apps allowed users to opt-out of data collection, and only 2 apps specified that they meet criteria for HIPAA (Health Insurance Portability and Accountability Act) compliance.

### Data Sharing

Most apps did not have functionality that allowed data sharing with others, such as clinical providers, to assist with clinical management. Less than 25% (50/228 in 2021 and 45/180 in 2022) of apps allowed the user to download or export data, and similarly few apps (<23%, 39/228 in 2021 and 42/180 in 2022) noted that users owned their own data. None of the rated apps connected to medical records for data sharing with providers.

### Evidence and Clinical Foundation

We found that all rated apps had clinical claims (meaning they claimed to assist with quitting smoking) and demonstrated face-validity (meaning they provided relevant content that was consistent with that goal). However, supporting published papers such as those describing feasibility or efficacy research were only available for 12 (5.2%) apps in 2021 and 11 (6.1%) apps in 2022.

### Intervention Features and Engagement Style

The most common intervention features were goal setting and habits (over 56%, 128/228 in 2021 and 115/180 in 2022) that allow the user to set and check in on goals toward quitting, followed by psychoeducation, such as providing information about nicotine, withdrawal, and craving (Table 2). More apps in 2022 included the ability to track symptoms (92/180, 51% vs 53/228, 23% in 2021). Very few apps included CBT (5/228, 2% in 2021 and 4/180, 2% in 2022). The most common app engagement tools included personalization with user-generated data (over 86%, 198/228 in 2021 and 157/180 in 2022), followed by gamification (over 42%, 97/228 in 2021 and 79/180 in 2022). Less common features included the ability to chat through message boards (36/228, 15% in 2021 and 28/180, 15% in 2022) or with peers (ie, someone with lived experience; 33/228, 15% in 2021 and 27/180, 15% in 2022).

**Table 2:** Commonly identified intervention features and app engagements.

Features			Apps in 2021 (N=228), n (%)		Apps in 2022 (N=180), n (%)
**Intervention features**					
	Goal setting and habits	128 (56.1)		115 (63.9)	
	Psychoeducation	106 (46.5)		59 (32.8)	
	Track symptoms	53 (23.2)		92 (51.1)	
	Productivity	47 (20.6)		19 (10.6)	
	Mindfulness	41 (18)		25 (13.9)	
	Journaling	40 (17.5)		35 (19.4)	
	Peer support	36 (15.8)		28 (15.6)	
	Track mood	26 (11.4)		19 (10.6)	
	Deep breathing	23 (10.1)		23 (12.8)	
	Coach or therapist connection	13 (5.7)		7 (3.9)	
	Chatbot interaction	11 (4.8)		10 (5.6)	
	Picture gallery or hope board	11 (4.8)		10 (5.6)	
	Physical health	7 (3.1)		5 (2.8)	
	Biodata	7 (3.1)		1 (0.6)	
	Track medication	5 (2.2)		3 (1.7)	
	Cognitive behavioral therapy	5 (2.2)		4 (2.2)	
	Exercise activities	5 (2.2)		2 (1.1)	
	Track sleep	1 (0.4)		0 (0)	
	Acceptance and commitment therapy	0 (0)		1 (0.6)	
	Dialectical behavior therapy	0 (0)		0 (0)	
**App engagements**					
	User-generated data	198 (86.8)		157 (87.2)	
	Gamification (eg, earn points and prizes)	97 (42.5)		79 (43.9)	
	Audio	55 (24.1)		40 (22.2)	
	Chat and message board	36 (15.8)		28 (15.6)	
	Realtime response to chats	33 (14.5)		7 (3.9)	
	Peer support communication	33 (14.5)		27 (15)	
	Video	20 (8.8)		16 (8.9)	
	AI^a^ support	13 (5.7)		8 (4.4)	
	Network support (eg, connect with others in own network like family and friends)	11 (4.8)		15 (8.3)	
	Screeners and assessments	7 (3.1)		7 (3.9)	
	Collaboration with provider	5 (2.2)		1 (0.6)	
	Asynchronous response to chats	1 (0.4)		5 (2.8)	

^a^AI: artificial intelligence.

## Discussion

### Overview

This study rated and reviewed smartphone apps available on the Apple and Android app store marketplaces related to smoking cessation using a systematic rating framework from the APA app evaluation model to evaluate app content. While efforts have been made to standardize approaches to reviewing apps (such as MARS and uMARS) [25], these systems are limited to testing app usability (ie, functionality), and rating scores are not often publicly available. The current study using the APA app rating system complements these efforts by providing a centralized, common app review approach that is accessible to the potential intended end users (ie, adults who smoke and want to quit) through a web-based and searchable database at mindapps. Our analyses of the top-returned apps indicate that while hundreds of apps exist in the marketplace for smoking cessation, many are nonfunctioning or irrelevant, most lack supporting clinical studies evaluating efficacy, and the availability of effective intervention features for smoking cessation is variable. These findings underscore the challenges for consumers to identify relevant, evidence-based apps to support their efforts to quit smoking.

Our results suggest that users need to be cautious in searching for smoking cessation apps since many apps were irrelevant or have the potential to pose risks related to data privacy and security. Specifically, 41% (160/389) of the initial apps returned in searching the app stores with common phrases for smoking and cigarettes were nonfunctioning or irrelevant, and rather than assisting in quitting, they have the potential to interfere with quitting, since many provided content to encourage or facilitate smoking (eg, simulate smoking, web-based purchasing of products, and finding places where smoking is allowed). Additionally, we reviewed the privacy policies and data sharing to evaluate potential privacy risks to consumers. Among the smoking apps identified, only 2 apps were HIPAA compliant, and at least half did not clarify the data use or purpose or describe any data security measures. Additionally, less than 12% (24/228 in 2021 and 17/180 in 2022) of apps had clearly defined processes that allowed users to opt out of data collection. This finding is unfortunately consistent with other research, and problems with data privacy and security have been noted for popular mental health apps [32,38]. Thus, the potential benefit of widely available consumer apps to help with quitting smoking is offset by concerns about risk due to the challenges of finding useful, functioning apps that protect user privacy.

Furthermore, another central challenge we identified is very few apps from the app store have published studies supporting their functionality or efficacy to help people quit smoking; for example, only 12 apps in 2021 and 11 apps in 2022 [11,16,39-44]. Other reviews have similarly noted that most apps have not been tested in clinical studies [8,17]. To evaluate the potential clinical use, we can also use the APA app rating system to examine whether the app is based on any theoretical foundation or evidence-based behavior change technique [8,24]. In our review of the top-returned apps, it is promising that most apps included goal-setting and psychoeducation; however, overall inclusion of features known to support health behavior change was low. For example, we found that less than 1 in 5 apps provided evidence-based support for smoking cessation, such as peer support or skill training, including mindfulness and deep breathing, and even fewer provided evidence-based approaches, such as Acceptance and Commitment Therapy or CBT interventions. Similar findings were noted in another recent review, where only 3 apps provided CBT skill training [45]. Skill training is a key component of clinical practice guidelines for effective smoking cessation treatment [46]. Thus, many of the top-returned apps for consumers in the app store likely do not provide essential skill training to support quitting smoking. It is also important to note that some apps that do have strong evidence supporting their efficacy may not appear in search results for consumers. For example, iCanQuit is an app with a strong evidence base from several studies demonstrating success in helping people quit smoking [16,18], but this app did not appear in the top 100 returned apps with the search terms used. Notably, 1 review found that evidence-based apps were returned in the app stores only when search terms used more formal language (ie, “smoking cessation” vs “quit smoking”) despite the fact that users are more likely to use plain language [9]. Thus, changes to the indexing and organization of apps may be needed to improve the ability of average consumers to find apps most in line with scientific evidence or underlying medical theory.

Additionally, the use of publicly available and searchable databases such as mindapps may be valuable for helping users find relevant apps. All app ratings used for the study were deposited in mindapps, so anyone can access and benefit from this information. Of note for this paper, the database is continuously reviewed and updated, which is essential since there is an incredibly high turnover of apps over time. In our analysis, we observed that approximately 68% (157/228) of apps that were available in July 2021 remained available in November 2022. Other studies have found similar high turnover in apps, with 1 study reporting that the rate of turnover in apps for depression was 1 app becoming unavailable every 2.9 days [47]. Volatility in the available apps creates problems for sustained use and suggests many apps are not maintained over time. Thus, having a publicly available rating system that is continuously updated, like the mindapps database, may be especially helpful for consumers to find relevant apps.

Findings should be considered in light of study limitations. Specifically, this study used 4 key search terms and examined the top-returned apps for these terms. We rated all top-returned apps that contained smoking cessation content and did not distinguish between apps designed specifically for smoking cessation (eg, “quit smoking”) and those that had a more general focus (eg, “meditation”) but provided smoking cessation information. We sought to analyze the apps most readily available to a layperson using these search terms; however, our search may have missed some relevant apps, and as noted above, some apps with strong scientific support may not readily appear in a search of the app store. Also, studies have documented variability and individual differences in algorithms that determine which apps appear in the app store [47]. Thus, this review is not an exhaustive review of all smoking cessation apps but aims to serve as a reference point on the current status of the field and notes key areas for further development that are needed to optimize the use of these apps for the public. We indicated which apps had published supporting studies at the time of data collection, although we did not evaluate the quality of the published evidence. Given the dynamic nature of the app store, it is likely that the list of available apps rapidly changes. Additionally, some data are not available for historical apps that are now defunct (eg, reading level). However, more detailed information about currently available apps and the rated features of these apps can be found at mindapps, which is updated every 6 months. Furthermore, very few studies have published papers on the feasibility or efficacy of the apps, so statistical comparisons between those with and without empirical support are limited, and this would be an important area for future research.

### Conclusions

Although hundreds of apps are available in a commercial marketplace when searching for support, quitting smoking, even in late 2022, there remains a challenge for finding an appropriate resource. Very few of the top-returned apps in the commercial marketplace provide evidence-based support, and many are irrelevant to quitting smoking. Also, some apps that are published in research reports showing clinical efficacy are not readily returned in a search on the app store. There is great potential for smartphone apps as digital tools to provide support for quitting smoking, yet advances are needed to close the gap between research and commercial access.

## Appendix

Multimedia Appendix 1 American Psychiatric Association (APA) App Evaluation Model Rating Questions and Descriptions.

Multimedia Appendix 2 Names of top-returned smoking cessation apps in 2021 and 2022.

## Abbreviations

APA: American Psychiatric Association
CBT: cognitive behavioral therapy
HIPAA: Health Insurance Portability and Accountability Act
MARS: Mobile App Rating Scale
uMARS: user version of the Mobile App Rating Scale

## Data Availability

The data sets generated and analyzed during this study are available from the corresponding author on reasonable request. All data related to ratings for smoking cessation apps are publicly available at mindapps website.
