# Supplementary material for: Smartphone Apps for Smoking Cessation: Systematic Framework for App Review and Analysis
Source: J Med Internet Res. 2023 Jul 13;25:e45183. doi: 10.2196/45183 (PMC10375280; doi:10.2196/45183)
Supplement: Multimedia Appendix 1 [file jmir_v25i1e45183_app1.pdf]

Appendix 2. APA App Evaluation Model Questions and Descriptions.

	Question	Description
	**App Origin**	
1	Does it come from the government?	Does the app come from a government agency? Examples include apps published by the Department of Defense and Veterans Administration.
2	Does it come from a for-profit company or developer?	Most apps on the app store come from a for-profit company or individual developer. If the name of the developer is followed by "LLC" assume it is for-profit.
3	Does it come from a non-profit company?	To determine if the app developer is a non-profit company, it may be helpful to refer to the company's website. An example of a non-profit developer is the Schizophrenia and Related Disorders Association of America, which developed the Schizophrenia Health Storylines App.
4	Does it come from a trusted healthcare company?	A hospital is an example of a healthcare developer. Some apps may be developed by a university-affiliated hospital, in which case the app comes from both a healthcare company and academic institution.
5	Does it come from an academic institution?	Apps that are developed by academic institutions include apps affiliated with universities (Harvard, Stanford, Dartmouth, UCSF NYU are just a few examples of academic institutions that have developed mental health apps).
	**App Functionality**	
6	Does it work on Apple(iOS)?	Does the app have an entry on the iOS app store?
7	What is the Apple version?	(from Apple store link)
8	What is the oldest iOS version supported?	(from Apple store link)
9	What was the Apple release date?	(from Apple store link)
10	When was the last Apple (IOS) update?	(from Apple store link)
11	Has the apple version been updated in the last 180 days?	(from Apple store link)
12	Number of reviews on Apple store?	(from Apple store link)
13	Rating (number of stars) on Apple store?	(from Apple store link)
14	App size on iOS?	(from Apple store link)
15	Does it work on Android?	Does the app have an entry on the Google Play Store?
16	What is the Android version?	(from Google Play store link)
17	What is the oldest Android version supported?	(from Google Play store link)
18	What was the Google play store release date?	(from Google Play store link)
19	When was the last Android update?	(from Google Play store link)
20	Has the android version been updated in the last 180 days?	(from Google Play store link)
21	Number of reviews on google play store?	(from Google Play store link)
22	Rating (number of stars) on google play store?	(from Google Play store link)
23	App size on android?	(from Google Play store link)
24	Does the app work offline?	Does the app work in airplane mode?
25	Does it have at least one accessibility feature (like adjust text size, text to voi	Does the app work with adjustable text size setting of the phone? Or text to voice features? Does the app have an internal setting for incresing text size?
26	Does it work with Spanish?	This information is available on the app store.
27	Does it work with a language other than English or Spanish?	This information is available on the app store.
28	Is the app totally free?	An app is totally free if there are no costs up front and no in-app purchases.
29	What is the cost up front?	Some apps may be free up front but have in-app purchases.
30	Are there in-app purchases?	Are there any functionalities of the app that are inaccessible without payment?
31	Is it a subscription (recurrent/monthly/annual)?	Does full use of the app depend upon a subscription?
	**Inputs & Outputs**	
32	Input: surveys?	Does the app enable a user to enter surveys such as mood or symptom surveys?
33	Input: Diary?	Does the app have a journaling, diary, or free writing feature?
34	Input: Geolocation?	Does the app enable location services from the phone?
35	Input: contact list?	Can a user connect their contact list to the app?
36	Input: Camera?	Do any features of the app utilize camera input? So profile picture? Or photo diary features? Or video chat?
37	Input: Microphone?	Does the app allow a user to record using the phone microphone?
38	Input: step count?	Does the app utilize step tracking?
39	Input: external devices (e.g. a wearable sending direct data)?	Does the app connect with an external device such as a smart watch or heart rate monitor?
40	Input: social network?	Connection to social media. Does the app allow you to input social media information? For example, do you connect it to your facebook to log in? Or do you connect with social media contacts through the app?
41	Output: notifications?	Does the app send notifications? These notifications could be incoming messages, reminders from the app, or alerts.

42	Output: psychoeducational references/information?	Does the app provide psychoeducational references or information? (Note: this means the exact same thing as the question about features: psychoeducation).
43	Output: social network?	Can you post information from the app to social media? Does the app connect to social media for posting purposes?
44	Output: reminders?	Does the app allow you to set reminders? (Oftentimes these reminders will then generate notifications)
45	Output: graphs of data?	Does the app allow a user to see graphically depicted data?
46	Output: summary of data (in text or numbers)?	Does the app provide written summaries of data (description of data apart from a graph)?
47	Output: link to formal care/coaching?	Does the app connect a user with a healthcare provider? A licensed therapist or clinician?
	**Privacy & Security**	
48	Is there a privacy policy?	The following questions involve reading the privacy policy, which will be linked to the app store if the app has one.
49	Does the app declare data use and purpose?	What information is the app collecting and for what purpose?
50	Does the app report security measures in place?	How is data protected? Does the app claim to collect and share data securely?
51	Is PHI shared?	PHI refers to personal health information that is entered into the app (name, birthday, content of messages, mental health information). Data is shared if it leaves the app in any way.
52	Is de-identified data shared?	De-identified data is information that has been stripped of personally identifiable attributes.
53	Is anonymized/aggregate data shared?	Most apps collect and share aggregate use data. This anonymized data has no traceable link to an individual.
54	Can you opt out of data collection?	Is there a way for a user to indicate that they don’t want to app to collect or share their data?
55	Can you delete your data?	Can user data be deleted? Some apps may retain data permanently.
56	Is the user data stored only on the device?	User data is either stored locally (exclusively on the device) or on a server.
57	Is the user data stored on a server?	If the data is stored only on the device, then it won’t be stored on a server.
58	Does the app have a crisis management feature?	The presence of a crisis management feature is often indicated in the privacy policy. A crisis management feature refers to an app’s emergency response: does the app provide a hotline number that can be called if user input suggests a crisis?
59	Does the app claim it meets HIPAA?	Does the app specify it is compliant with HIPAA and that health information is protected compliant with HIPAA standards?
60	Reading level of the privacy policy (what grade reading level)?	Flesch-Kincaid reading grade level: https://readabilityformulas.com/free-readability-formula-tests.php (just copy and paste privacy policy in).
61	Does the app use 3rd party vendors (i.e. google analytics, etc)?	Does the app mention use of third parties?
	**Evidence & Clinical Foundation**	
62	Does the app appear to do what it claims to do?	If the app claims to offer CBT, for example, is there evidence that CBT is provided on the app?
63	Is the app patient facing?	This will depend on the terms of the search. Is the app relevant for an individual with the condition specified in the search? Is it intended for patient use (some apps, for example, may be diagnosis guides and intended for use by healthcare providers but not patients).
64	How many supporting studies have been published? What is the highest imp	How many studies about the feasibility of this specific app’s use have been published? How many studies about the efficacy of this app to accomplish what it claims to do have been published? What is the impact factor of the journal in which the study is published? This can easily be found with a quick google search. If it's not immediately evident, put "0" (assume the journal doesn't have an impact factor.
65	Can the app cause harm?	Does the app make recommendations or suggestions that directly defy clinical guidelines? Does it include overtly false information, like a suicide hotline number that doesn't actually work?
66	Does the app provide any warning for use?	Is there any warning for a user that the app is not intended to replace medical care?
	**Features & Engagement Style**	
67	Features: mood tracking?	Does the app provide surveys where a user can enter their mood data?
68	Features: medication tracking?	Designated medication tracking feature?
69	Features: sleep tracking?	Does the app track sleep, either in conjunction with a wearable or through user-entered information?
70	Features: physical exercise tracking?	Does it allow a user to track duration or content of physical exercise?
71	Features: psychoeducation?	Does it provide definitions, explanations of different diagnoses? Is it didactic?
72	Features: journaling?	Is there a place for the user to journal or free write?
73	Features: picture gallery/hope board?	Does the app allow a user to curate a gallery of saved and searched images and quotes?
74	Features: mindfulness?	Any mindfulness exercises? May include deep breathing but not necessarily.
75	Features: deep breathing?	Does the app offer exercises in deep breathing?
76	Features: iCBT or sleep therapy?	Doe the app offer sleep therapy of any kind (including iCBT, a targeted sleep intervention)?
77	Features: CBT?	Does the app provide cognitive-behavioral therapy?
78	Features: ACT?	Does the app provide Acceptance and Commitment Therapy?
79	Features: DBT?	Does the app provide dialectical behavior therapy?

80	Features: peer support?	Does the app offer connection to peer specialists or individuals with lived experience?
81	Features: connection to coach/therapist?	The app has a built-in way to connect with a provider or coach.
82	Features: biodata?	Does the app collect heart rate or skin conductance?
83	Features: goal setting/habits?	Does the app allow users to set and check in on goals?
84	Features: productivity?	Does the app provide activities/advice for users to do daily tasks, related to goal setting and habits?
85	Features: physical health exercises?	Something like 7 minute workout that actually gives a workout (this is a recommendation of exercises, NOT tracking.
86	Features: Bbot interaction (like with virtual character)?	The app allows a user to interact with a virtual character.
87	Features: Biofeedback with sense data (EEG, HRV, skin conductance, etc)?	The app uses biodata to provide feedback/recommendations (an app that will recommend more breathing exercises to respond to high heart rate, for example).
88	Engagement style: user generated data?	Does the user engage with the app by inputting their own data (for example, mood tracking or diary)?
89	Engagement style: chat/message based?	User can send and receive messages.
90	Engagement style: is it a screener/assessment?	Examples include PHQ9, GAS7, etc.
91	Engagement style: real time response?	Someone will reply to your chat right away.
92	Engagement style: Asynchronous response?	There are no immediate responses to chats; responses come at predetermined intervals (once a day; every four hours; etc)
93	Engagement style: gamification (points, badges)?	User can win points and prizes for engaging with the app.
94	Engagement style: videos?	App includes videos user can view.
95	Engagement style: audio/music/scripts?	Does the app provide music or audio experiences? Some meditation apps, for example, utilize audio sessions.
96	Engagement style: AI support?	Interaction is not with a real person but with a bot.
97	Engagement style: peer support?	Peer is defined as a person with lived experience and support involves actually communcating (so not just watching a video).
98	Engagement style: network support?	Network is defined as someone (like family or friend) who is actually known.by the user outside the app. An example is an app that allows a user to communicate with family members about relevant health information.
99	Engagement style: Collaborative with provider/other?	Does it allow for direct collaboration with a provider or clinician? Beyond just being able to share your data.
	**App Use**	
100	Is it a self-help/self-management tool?	Provides activities that can be used for self-help and self-management, such as mood or symptom tracking or mindfulness exercises.
101	Is it a reference app?	Provides information and references but not necessarily activities. psychoeducation first.
102	Is it intended for hybrid use with a clinician in conjunction with treatment plan?	Is the app intended to be used as an adjunct to care? Apps that have built-in methods of connecting with a provider meet this criteria. However, a teletherapy app would not be intended for hybrid care, as the app replaces in-person care.
	**Interoperability & Data Sharing**	
103	Do you own your data?	This can be found in the privacy policy of the app but is relevant for data sharing capacity. Can a user see and access their data from the app?
104	Can you email or export your data?	Can data be downloaded or exported, or emailed straight from the app?
105	Can you send your data to a medical record?	Does the app sync with EMR? (most apps are not currently equipped with connections to medical record, so this will likely be no)

Note: APA App Evaluation Model terms and framework can be found here: https://mindapps.org/FrameworkQuestions
