# Supplementary material for: Smartphone Apps for Smoking Cessation: Systematic Framework for App Review and Analysis
Source: J Med Internet Res. 2023 Jul 13;25:e45183. doi: 10.2196/45183 (PMC10375280; doi:10.2196/45183)
Supplement: Multimedia Appendix 2 [file jmir_v25i1e45183_app2.pdf]

Appendix 1

App Name 2021 data (N=228)	App Name 2022 data (N=180)
Adiquit: Quit Smoking	A New Pair of Glasses 12 Steps
Aeris: Quit Smoking & Vaping - Free Tracker App	Adiquit: Quit smoking
Alex AI - Quit Smoking	Aeris: Quit Smoking & Vaping - Free Tracker App
AshTray - Cigarette Counter	Alanon Workshops AA 12 Steps
Beat Smoking - Quit Smoking	Alex AI -Quit smoking
Break Free Rocket	appy - stay sober today
Break if Off - Quit Smoking	AshTray - Cigarette Counter
Breathe Easy Smoking Cessation	Beat Smoking - Quit Smoking
Breathe Now - Stop smoking	Become a non-smoker now
Butt Out Quit Smoking Forever	Benji Â· Quit Habits Tracker
Chantix - Quit Smoking	Breathe Easy Smoking Cessation
Cigano: Quit Smoking Challenge	Breathe Now - Stop smoking
Cigared - Smoke Counter	Butt Out Quit Smoking Forever
Cigarette Analytics	Chantix - Quit smoking
Cigarette Book	Cigano: Quit Smoking Challenge
Cigarette Calculator	Cigarette Analytics
Cigarette Control & Counter	Cigarette Book
Cigarette Count	Cigarette calculator
Cigarette Counter	Cigarette Control & Counter
Cigarette counter	Cigarette Count
Cigarette Counter - How much do you smoke?	Cigarette Counter
Cigarette Counter / Quit Smoking / Don't smoke	Cigarette counter
Cigarette Counter and Tracker	Cigarette Counter - How much do you smoke?
Cigarette Counter Lite - How much do you smoke?	Cigarette Counter and Tracker
Cigarette Tracker App	Cigarette Counter Lite - How much do you smoke?
Cigarettes Control (Pro)	Cigarette Tracker App
Cigarettes Counter	Cigarettes Control (Pro)
Cigarettes Lite	CiggyCount - Watch your smokes
CiggyCount - Watch your smokes	Cigs Counter
Cigs Counter	Cigs Counter - Quit Smoking
Cigs Counter - Quit Smoking	Clean Day - Sobriety Counter
Clean Time App	Counter Tally Count
Coffee & cigarettes	*Craving To Quit!
Count my cigarettes	Darth Vaper
Counter – Tally Counter	Days Since: Quit Habit Tracker
*Craving To Quit!	DipQuit Pro: Quit Dipping Smokeless Tobacco
Darth Vaper	Done: A Simple Habit Tracker
Days Since: Quit Habit Tracker	Don’t Smoke: 30 Days Challenge

Dependn' – Quit weed, tobacco and alcohol – Stop drinking & smoking	Drop It! Quit Smoking
Done: A Simple Habit Tracker	DWS: Smoke-free counter
Don't Smoke: 30 Days Challenge	Easy Quit Smoking & Vaping
Drop it! Quit Smoking	Empowered Hypnosis for Alcoholism & Addiction
DWS: Smoke-free counter / Quit Smoking Now	Enjoy! Quit Smoking
Easy Quit Smoking & Vaping	ExSmoker – Stop Smoking Now
Easy Way to stop smoking	Flamy – quit smoking
Enjoy! Quit Smoking	Fumolite
ExSmoker – Stop Smoking Now	Get Rich or Die Smoking Gold
Flamy – quit smoking & become a non-smoker	Habit Tracker ++
Fumolite	Hypnosis for Quit Smoking
Get Rich or Die Smoking Gold	I Can – Sober Counter
Habit Tracker ++	I Give Up Smoking
Healthy Quit	IcanCaRe – Online Tobacco Wellness App
How to Quit Smoking Breaking Bad Habits	ISMOKAY – QUIT SMOKING
HypnoCloud | Hypnotherapy App	iSmoke2Much
Hypnomatic — mobile hypnosis	JustQuit: Tracker for Recovery
Hypnosis for Quitting Smoking Guide Free	*Kwit - Quit smoking for good!
I Am Sober	Last : Track Time Passed Since
I Can – Sober Counter	Last Cigarette
I Give Up Smoking	LIVESTRONG MyQuit Coach
ICanCaRe - Online Tobacco Wellness App	My Last Cigarette
ISMOKAY - QUIT SMOKING	My last Cigarette Timer
iSmoke2Much	Never Better
KickSmoke: quit smoking now!	No Smoking
*Kwit - Quit Smoking for good!	No-Cotine! Quit smoking
Last : Track Time Passed Since	nomo - Sobriety Clocks
Last Cigarette	NoSmokingWatch-Decided version of non-smoking app!
Last Cigarette	Puff Count
LIVESTRONG MyQuit Coach	Quit Anything - X days since
My Last Cigarette	Quit Cigs, Smoking, & Tobacco - Quit Smoking Now
My last Cigarette Timer	Quit It - stop smoking today
MyQuit	Quit it - stop smoking today
No Smoking	*Quit Now: My QuitBuddy
No Smoking - The Best App to Quit Smoking	Quit Right - Quit Smoking Now - Stop Smoking
No-Cotine! Quit Smoking	Quit Smoking
Nomo - Sobriety Clocks	Quit Smoking
NoSmokingWatch-Decided version of non-smoking app	Quit smoking
Productive - Habit Tracker	Quit Smoking

Queste: quit smoking now	Quit Smoking - Get Smoke Free
Quit Cigs, Smoking, & Tobacco - Quit Smoking Now	*Quit Smoking - MindCotine
Quit For Health	Quit Smoking - Premium Subliminal Messages
Quit it - stop smoking today	Quit smoking - Smokerstop
Quit It - stop smoking today	Quit Smoking - Stop Smoking Counter
Quit My Way	Quit Smoking - Stop Smoking Now
*Quit Now: My QuitBuddy	Quit Smoking - Stop Smoking without any medicine
Quit Pro	Quit Smoking - We are your motivation
Quit Right - Quit Smoking Now - Stop Smoking	Quit Smoking (Lite)
Quit Smoking	Quit Smoking
Quit Smoking	Quit Smoking Audiobook
Quit Smoking	Quit smoking cigarettes with Smokler
Quit Smoking	Quit Smoking Get Healthy
Quit Smoking	Quit Smoking Hypnosis - Stop Smoking Hypnotherapy
Quit Smoking	Quit smoking in 101 days
quit smoking	Quit Smoking -No smoking day
Quit Smoking - Get Smoke Free	*Quit Smoking NOW - Max Kirsten
*Quit Smoking - MindCotine	Quit smoking now! - Tobakko
Quit Smoking - No smoking day	Quit Smoking Now: Quit Buddy!
Quit Smoking - Premium Subliminal Messages	Quit Smoking Slowly - Gradually
Quit Smoking - Smokerstop	Quit Smoking Timer - Smoke less, quit your habit!
Quit Smoking - Stop Smoking Counter	Quit Smoking Today - quitcy
Quit Smoking - Stop Smoking Now	Quit Smoking Tracker
Quit Smoking - Stop Smoking without any medicine (Early Access)	Quit Smoking Tracker
Quit Smoking - Stop Tobacco Mobile Trainer	Quit Smoking Tracker GOLD - stop smoking app
Quit Smoking - We are your motivation	Quit Smoking Virtual
Quit Smoking (Lite)	Quit Smoking with AJ
Quit Smoking --~	Quit Smoking with Andrew Johnson
Quit Smoking 30 days Plan: Stop Smoking Tracker	Quit Smoking With Hypnosis
Quit Smoking Affirmations	Quit smoking with Quitify PRO
Quit Smoking Audiobook	Quit Smoking, Be Smoke Free
Quit Smoking Cigarettes with Smokler	Quit Smoking-App : Stop Smoking Cigarettes
Quit Smoking Forever - EFT	Quit Tobacco
Quit Smoking Get Healthy	Quit Tracker: Stop Smoking
Quit Smoking Hypnosis - Stop Smoking Hypnotherapy	Quit Vaping
Quit Smoking in 101 days	Quit: Hypnosis Program to Stop Smoking
Quit Smoking in 28 Days Audio Program	*QuitGuide - Quit Smoking
*Quit Smoking NOW - Max Kirsten	QuitNow: Quit smoking for good

Quit smoking now – smoke free	Quitsmoke - Easily stop smoking
Quit Smoking Now: Quit Buddy	QuitSmoke - Quit Smoking Now
Quit Smoking Pro	*quitSTART - Quit Smoking
Quit Smoking Self Hypnosis	QuitSure: Quit Smoking Smartly
Quit Smoking Slowly - Gradually	Quitter - Daily Habit Tracker
Quit Smoking Timer - Smoke less, quit your habit!	Quitzilla: Bad Habit Tracker
Quit Smoking Today - quitcy	Qwit (Quit Smoking)
Quit Smoking Tracker	Redlight quit Smoking
Quit Smoking Tracker	Reveri
Quit Smoking Tracker GOLD - stop smoking app	Since - Day Counter & Tracker
Quit Smoking Virtual	Since iQuit
Quit Smoking with AJ	*SiS - Smiling Instead of Smoking
Quit Smoking with Andrew Johnson	Smoke - quit
Quit Smoking with Hypnosis	Smoke - quit Pro
Quit Smoking with Quitify PRO	Smoke diary - cigarette counter
Quit Smoking, Be Smoke Free	Smoke Finance
Quit Smoking, NeverSmoking	Smoke FREE - Non Smoking
Quit smoking. No bad Habits	*Smoke Free - quit smoking now
Quit Smoking. Stop Vaping Now	Smoke Free App
Quit Smoking-App : Stop Smoking Cigarettes	Smoke Free: Stop, Quit, No Smoking - Quit Tracker
Quit Tobacco	Smoke Meter
Quit Tobacco	Smoke Out: Tobacco Pirates
Quit Tracker: Stop Smoking	Smoke Timer - Quit Smoking
Quit: Hypnosis Program to Stop Smoking	Smoke Watchers - Quit smoking
*QuitGuide - Quit Smoking	SmokeFree - quit smoking slowly
QuitNow! PRO - Stop Smoking	Smokefree 2 - Quit Smoking
QuitNow! Quit Smoking	Smokeless: Reduce or quit
Quitos - Quit Smoking	Smokenote Pro - Quit Smoking
Quitsmoke - Easily stop smoking	SmokeQuitter - The No-nonsense Cigarette iQuit App
QuitSmoke - Quit Smoking Now	*Smokerface
*quitSTART - Quit Smoking	Smokers Diary
QuitSure - Quit Smoking Smartly. No Cravings	Smoking cessation Quit now Stop smoke hypnosis app
Quitzilla	Smoking Counter & Stats
Qwidder: Quit Smoking	Smoking Less
Qwit (Quit Smoking)	Smoking Log
Redlights quit Smoking	Smoking Log - Stop Smoking
Reduce and Stop Smoking	Smoking Log Plus License
Reveri	Smoking manager - Smokegram
Self discipline for willpower	Smoking Note
Sharecare: Health & Well-being	Smokitten - Quit smoking !

Since - Day Counter & Tracker	SmoQuit - quit smoking
*SiS - Smiling Instead of Smoking	Sober Time - Sober Day Counter
Smoke - quit	SoberBuddy: Addiction Recovery
Smoke - quit Pro	SoberTool - Addiction Help
Smoke - quit Pro	Sobriety Tracker Counter App
Smoke diary - Cigarette Counter	*Stay Quit Coach
Smoke Finance	Stop Cigarette
Smoke FREE - Non Smoking	Stop Smoking - EasyQuit
Smoke FREE - quit smoking Plus	Stop Smoking - EasyQuit Pro
Smoke Free App	Stop smoking - is an easy way to give up smoking
*Smoke Free: Quit Smoking Now and Stop for good	Stop Smoking - Quit Smoking
Smoke Free: Stop, Quit, No Smoking - Quit Tracker	Stop Smoking - quit smoking, be smoke free
Smoke Meter	Stop Smoking Cessation Tracker
Smoke Out: Tobacco Pirates	Stop Smoking Cigarette Tracker
Smoke Revoke - Gradually Quit Smoking	Stop Smoking Easy
Smoke Timer - Quit Smoking	Stop Smoking in Two Weeks - With Hypnosis!
Smoke Timer - Quite Smoking	Stop Smoking With Hypnosis Expert James Holmes
Smoke Watchers - Quit Smoking	Stop Smoking: Simple Helper
Smokefree - Quit smoking	Stop Tobacco Mobile Trainer
SmokeFree - quit smoking slowly	SWay: Quit or Less Smoking Timer Cigarette Tracker
SmokeFree Pro	Tobacco - Quit Smoking
Smokeless: Reduce or quit smoking	Tobacco Free Teens
Smokenote Pro - Quit Smoking	Tobacco Quiz
SmokeQuitter - The No-nonsense Cigarette iQuit App	Tobano - quit smoking
Smoker Insight! -- A Smoker's LifeStyle Analyzer	You Can Quit Smoking
*Smokerface	
Smokers Diary	
Smoker's diary	
*smokeSCREEN game	
Smoking Cessation Hypnosis	
Smoking cessation Quit now Stop smoke hypnosis app	
Smoking Counter & Stats	
Smoking Less	
Smoking Log	
Smoking Log - Stop Smoking	
Smoking Log Plus License - Stop Smoking	
Smoking Manager - Smokegram	
Smoking Note	
Smokitten - Quit Smoking	

SmoQuit - quit smoking	
Sobriety Tracker Counter App	
*Stay Quit Coach Legacy	
Stop Cigarette	
Stop Smoking - EasyQuit free	
Stop Smoking - EasyQuit Pro	
Stop smoking - is an easy way to give up smoking	
Stop Smoking - Quit Smoking	
Stop Smoking - Quit Smoking & Healthy Life	
Stop Smoking - Quit Smoking Forever	
Stop Smoking - Quit Smoking Tracker	
Stop Smoking - quit smoking, be smoke free	
Stop Smoking / Quit Smoking / Quit Tracker	
Stop Smoking app - Quit Cigarette and Smoke Free	
Stop Smoking Easy	
Stop Smoking Hypnosis	
Stop Smoking in Two Weeks - With Hypnosis!	
Stop smoking My quit coach app	
Stop Smoking Personal Stories of Success Quit Now	
Stop Smoking with Hypnosis Expert James Holmes	
Stop Smoking: Simple Helper	
StopSmoking - Quit cigarette smoking habits	
Streaks	
Streaks for Small Starts — Create healthy habits	
SWay: Quit or Less Smoking Timer Cigarette Tracker	
tabac.io	
Tobacco - Quit Smoking	
Tobacco Free Teens	
Tobacco Minus	
Tobacco Quiz	
Tobano - quit smoking	
Well - Feel Better	
Without smoking	
You Can Quit Smoking	

Note: * indicates app had published supporting study at the time of data collection.
